# The Impact of the COVID-19 Pandemic on the Severity of Alcohol Use Disorder: Significance of Dual Disorders

**DOI:** 10.3390/ijerph20206939

**Published:** 2023-10-19

**Authors:** Janka Gajdics, Otília Bagi, Fanni Fruzsina Farkas, Bálint Andó, Ildikó Katalin Pribék, Bence András Lázár

**Affiliations:** Addiction Research Group, Department of Psychiatry, University of Szeged, 6720 Szeged, Hungary; jankavongajdics@gmail.com (J.G.); bagiiotilia@gmail.com (O.B.); ffannif24@gmail.com (F.F.F.); ando.balint@med.u-szeged.hu (B.A.); ildikopribek@gmail.com (I.K.P.)

**Keywords:** COVID-19 pandemic, alcohol use disorder, delirium tremens, psychiatric co-morbidity, dual disorders

## Abstract

The coronavirus disease 2019 (COVID-19) pandemic has been assumed to impact patients diagnosed with alcohol use disorder (AUD). The severity of the influence that the COVID-19 pandemic had on the symptoms of AUD has not yet been revealed in detail. The aim of this study was to examine the impact of the COVID-19 pandemic on patients diagnosed with AUD. This retrospective study was conducted between 11 March 2017 and 31 May 2022 in Hungary. Medical charts (N = 1082) of inpatients with the diagnosis of AUD were reviewed. Based on the dates of admissions, two groups were created: the ‘before COVID-19’ group (11 March 2017–10 March 2020) and the ‘during COVID-19’ group (11 March 2020–31 May 2022). Chi-square tests, independent-sample t-tests, and multinomial logistic regressions were performed. The occurrence of delirium tremens (DT) and psychiatric co-morbidities was significantly higher during the pandemic. Our results showed that the occurrence of DT and psychiatric co-morbidities significantly increased during the pandemic. Our results revealed that the pandemic enhanced the severe consequences of AUD, and the development of AUD might have increased in frequency among individuals previously diagnosed with mental illness during the pandemic. These findings indicate the significance of dual disorders in the post-pandemic period.

## 1. Introduction

The coronavirus disease 2019 (COVID-19) pandemic caused by severe acute respiratory syndrome coronavirus 2 (SARS-CoV-2) is one of the most significant health care problems of the 21st century. As of July 2023, the COVID-19 pandemic has been responsible for more than 7 million deaths worldwide [1]. In late December 2019, the China Health Authority reported four cases of severe pneumonia of unknown aetiology in Wuhan. Later, a novel enveloped-RNA betacoronavirus was isolated from throat swab samples of patients, and, due to the rapid spreading of the illness, it was declared a pandemic by the World Health Organization on 11 March 2020 [2,3].

Although there were two distinct eras in terms of vaccine availability during the first two years of the pandemic, at the beginning and during the pandemic, the defence against the pathogen relied mainly on restrictions. These protective measures were effective; however, they had various psychological, social, and economic consequences [2]. The pandemic was associated with social isolation, economic difficulties, uncertainty, fear, and anxiety [4,5,6].

These factors have an important role in the development and worsening of psychiatric disorders. Several reports have revealed that psychosocial issues during the pandemic led to a relapse or worsening of substance use, depression, anxiety, and other mental disorders [5,6,7,8,9]. Furthermore, a negative impact of the COVID-19 pandemic on individuals with dual disorders (DDs) has also been suggested [6,10].

DDs are special conditions where mental illnesses occur along with substance use problems or disorders [10]. These conditions are associated with a high rate of substance use relapses, emergency admissions, and premature deaths [10,11,12,13,14]. Although the management of patients with DDs has a critical impact on mental health care, the diagnosis and treatment of these disorders suffer from various limitations due to a lack of data.

Recent studies have indicated that 20–75% of patients suffering from severe mental disorders have substance use disorders (SUDs) [15,16,17,18,19,20,21]. It has also been estimated that approximately 30% of these patients are polydrug users [22]. Additionally, it has been demonstrated that alcohol use disorder (AUD) is one of the most common types of co-morbid SUDs among patients diagnosed with psychiatric illnesses [15,16,17,18,19,20,21]. However, the role of the COVID-19 pandemic in the onset or worsening of DDs has not yet been clearly assessed.

Previously, various reports have examined the changes in SUDs during the pandemic. The majority of articles focused on the changes in alcohol consumption in the general population. Several studies found evidence of increased alcohol consumption during the COVID-19 pandemic [23,24,25,26,27]. In the summer of 2020, alcohol use started to increase after it became certain that the lockdown measures would continue [28]. In addition to younger age and male sex, job loss due to the pandemic was associated primarily with increased high-risk drinking. Stay-at-home restrictions, work-from-home responsibilities, financial burdens, social isolation, limited knowledge about the virus, and other uncertainties were also general causes of greater alcohol consumption [28,29]. In addition, patients have been shown to have increased treatment needs related to alcohol problems during the pandemic [23,27]. Thus, it was recommended that health authorities pay more attention to SUDs during and after the pandemic [30].

AUD—as one of the most common forms of SUDs both in the general population and in individuals diagnosed with psychiatric disorders—can lead to severe consequences such as alcohol withdrawal syndrome (AWS) and delirium tremens (DT) [31,32,33]. AWS includes most inpatient admissions of patients with AUD. AWS occurs when individuals with AUD suddenly stop or reduce their alcohol intake. Most patients will only develop uncomplicated or mild symptoms. However, about 20% of AWS is complicated and patients suffer from alcohol-related seizures and/or symptoms of DT [34]. DT occurs in approximately 5–20% of patients suffering from AWS, and the mortality rate is about 5% [31,35]. During the past few decades, several factors have been revealed as predictors for developing DT, such as somatic co-morbidities, elevated liver enzymes, and the number of previous DT episodes [31,32]. Although the occurrence of DT indicates the severity of the AUD, and it is known that alcohol consumption increased during the COVID-19 pandemic, the association between the pandemic and the occurrence of DT in patients diagnosed with alcohol dependence has not yet been studied in a clinical setting.

In summary, the COVID-19 pandemic had many effects on the patterns of alcohol consumption, the occurrence of psychiatric symptoms, and the health care system. These changes were mediated by both the direct and indirect effects of the pandemic, making it difficult to assess a direct causal relationship between the pandemic and its consequences. However, it is important to conduct further research in order to examine what changes occurred as a result of the pandemic in relation to AUD.

Therefore, the main goal of the present study was to evaluate the impact of the COVID-19 pandemic on the severity of the consequences on AUD during treatment by comparing clinical cases before and during the pandemic, and to assess the interplay between the COVID-19 pandemic, the secondary co-occurring psychiatric disorders, and DT.

## 2. Materials and Methods

### 2.1. Setting

The study was carried out with the aim of examining 1082 medical charts of 697 inpatients with a primary diagnosis of alcohol dependency syndrome (AUD; F10.2), alcohol withdrawal state (AWS; F10.3), or alcohol withdrawal state with delirium (DT; F10.4), according to the International Classification of Diseases, Tenth Revision (ICD-10) at the Department of Psychiatry, University of Szeged, Hungary, between 11 March 2017 and 31 May 2022. In this study, our main aim was to examine the impact of the pandemic on health care and the health condition of patients with AUD during their treatment. Since medical charts were analysed, there were individual patients who were admitted several times in this period (n = 54). Medical charts with the diagnoses of “Mental and behavioural disorders due to psychoactive substance use (F10–F19)” were excluded except for the diagnoses of F10.2, F.10.3, and F.10.4. The diagnoses were carried out by a consultant psychiatrist according to the AUD diagnostic criteria. The present work is a part of a larger study.

Two groups were created: inpatient admissions that occurred between 11 March 2017 and 10 March 2020 were the ‘before COVID-19’ group (N = 753), and those admissions that occurred between 11 March 2020 and 31 May 2022 were the ‘during COVID-19’ group (N = 329). These exact dates were chosen because 11 March 2020 was when the COVID-19 pandemic was announced, and 1 June 2022 was when the end of the pandemic was declared in Hungary. Considering that the legally defined period for the pandemic was longer than two years, we decided that the three years before the pandemic should be considered the ‘before COVID’ period.

Demographic variables (age and sex), somatic co-morbidities, levels of electrolytes (sodium and potassium) and liver enzymes [gamma-glutamyl transferase (GGT), serum glutamic-oxaloacetic transaminase (SGOT), serum glutamic pyruvic transaminase (SGPT), and SGOT/SGPT quotient], and platelet number were collected from the medical charts of inpatient admissions. Although total bilirubin and/or prothrombin time and partial thromboplastin time are markers of liver function used to determine the severity of liver injuries, previous reports revealed that the disturbances to transaminases could be risk factors for developing severe AWS [36,37]. Therefore, these variables were collected and analysed in the present study.

Secondary psychiatric diagnoses were also collected. AUD was the primary diagnosis in every case; secondary psychiatric diagnoses were determined by the consultant psychiatrist. However, due to the retrospective nature of the study, detailed features of the co-occurring psychiatric disorders were unknown.

The study was performed in line with the principles of the Declaration of Helsinki and was approved by the Human Investigation Review Board, University of Szeged (ethical approval number: 82/2022-SZTE). Due to the ethical guidelines of our Committee and the retrospective nature of the study, informed consent of the patients was not required because the study analysed anonymous clinical data of the patients.

### 2.2. Statistical Analysis

All statistical analyses were performed using IBM SPSS 24 [38].

Chi-square and independent-sample t-tests were used to compare demographic variables, somatic and psychiatric co-morbidities, and levels of electrolytes and liver enzymes between the two periods. In order to control biases caused by the unequal sample sizes, we calculated adjusted standardised residuals for the chi-square tests. Multinomial logistic regression models were used to determine the variables and the interactions between those variables that had an effect on admissions during the COVID-19 pandemic. To further investigate the role of these variables in patients’ admission during the COVID-19 time period, a second multinomial logistic regression model was used to examine the interactions between them. The dependent variable was the COVID-19 pandemic period; the independent variables were those that showed a significant difference between the two groups according to the previous analyses. *p* < 0.05 was considered statistically significant.

## 3. Results

### 3.1. Sample Characteristics

A total of 1082 medical charts of 697 inpatients were reviewed. There were 208 (18.75%) female and 874 (81.25%) male admissions in the sample, and their mean age was 51.42 (SD = 11.781). Approximately 69.6% (N = 753) of appearances occurred before the COVID-19 pandemic, while 30.4% (N = 329) of appearances occurred during the COVID-19 pandemic.

### 3.2. Characteristics of Admissions before and during COVID-19 Pandemic

The mean age of the ‘during COVID-19’ group (M = 52.5, SD = 11.567) was significantly higher than that of the ‘before COVID-19’ group (M = 50.92, SD = 11.853) (t(1038) = −2.020, *p* = 0.044). The ratio of female admissions in the ‘during COVID-19’ group was 21.3%, while, in the ‘before COVID-19’ group, it was 18.3%; however, there were no significant differences between the two groups (χ^2^ = 1.283, *p* = 0.257, OR = 1.204). The appearance of DT was significantly higher in the ‘during COVID-19’ group (13.1%) than in the ‘before COVID-19’ group (7.2%) (χ^2^ = 9.513, *p* = 0.002, OR = 1.946). The ratio of psychiatric co-morbidities was significantly higher in the ‘during COVID-19’ group (63.8%) than in the ‘before COVID-19’ group (48.1%) (χ^2^ = 22.342, *p* < 0.001, OR = 1.904). Table 1 shows the summarised characteristics of the two groups (Table 1).

### 3.3. Distribution Pattern of Psychiatric Co-Morbidities

**Figure 1:**
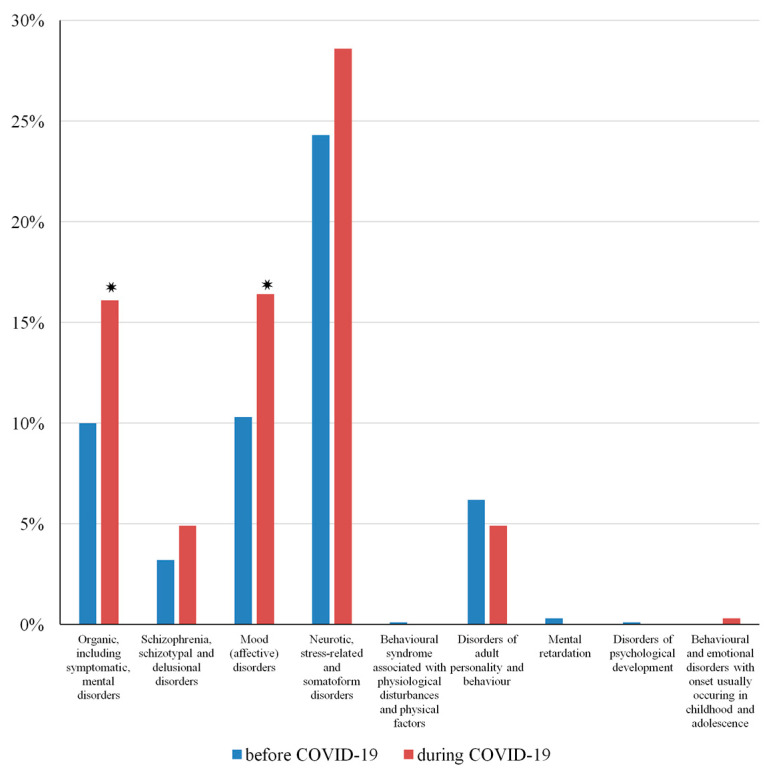
Distribution pattern of secondary psychiatric diagnoses in medical charts with a primary diagnosis of alcohol use disorder. Abbreviations: * = *p* < 0.05.

To reveal the impact of the pandemic on the onset of DDs, secondary psychiatric diagnoses were collected from the medical charts of inpatient admissions. Subgroups of diagnoses were created according to the ICD-10. The ratio of “Organic, including symptomatic, mental disorders (F00–F09)” was significantly higher in the ’during COVID-19’ (16.1%) group compared to the ’before COVID-19’ group (10%) (χ^2^ = 8.031, *p* = 0.005, OR = 1.731). Moreover, the ratio of “Mood (affective) disorders (F30–F39)” was significantly higher in the ’during COVID-19’ (16.4%) group compared to the ’before COVID-19’ group (10.3%) (χ^2^ = 7.926, *p* = 0.005, OR = 1.716). Furthermore, the ratio of “Neurotic, stress-related, and somatoform disorders (F40–F48)” was higher in the ’during COVID-19’ (28.6%) group compared to the ’before COVID-19’ group (24.3%); however, there were no significant differences between the two groups (χ^2^ = 2.190, *p* = 0.139, OR = 1.246). Figure 1 shows the distribution pattern of psychiatric co-morbidities (Figure 1).

### 3.4. Contributing Factors to Admissions during COVID-19

To determine which variables play a significant role in admissions during the pandemic, a multinomial regression model was constructed. The model consisted of variables that showed a significant difference between the ‘during COVID-19’ and ‘before COVID-19’ groups. Thus, age, DT, and psychiatric co-morbidity were included in the model, which was significant (χ^2^ = 29.570, *p* < 0.001) and had a 68.6% certainty. The occurrence of DT or a psychiatric co-morbidity significantly increased the likelihood of admission during the pandemic. Table 2 shows the results of the regression model (Table 2).

Another multinomial regression model was created focusing on the interactions of the above-mentioned variables. The model was significant (χ^2^ = 32.263, *p* < 0.001) and had a 68.8% certainty. The interaction of DT and psychiatric co-morbidity increased, while the interaction of DT and no psychiatric co-morbidity and the interaction of psychiatric co-morbidity and no DT decreased the probability of admission during the pandemic. Table 3 shows the results of the regression model (Table 3).

## 4. Discussion

To the best of our knowledge, this is the first study to assess the impact of the COVID-19 pandemic on the severity of AUD. The present data demonstrate that the occurrence of DT, the most severe consequence of AUD, was significantly higher during the pandemic. This result may suggest the presence of changes in the patterns of alcohol consumption during the pandemic. However, these results also support the fact that hospital admission criteria were different and rather focused on severe cases during the lockdown periods. Furthermore, in accordance with recent studies, our present findings may suggest that the COVID-19 pandemic had an effect on the onset of DDs and on the mental state of individuals previously diagnosed with psychiatric disorders. A regression analysis on the interactions of DT and psychiatric co-morbidities was also conducted to further investigate the role of these variables in patients’ admission during COVID-19. By performing the interaction analyses, we found out that delirium tremens and psychiatric co-morbidities did not only affect admissions in the COVID-19 pandemic period individually, but they also had a synergistic effect. This idea is supported by the result that the interaction of no delirium tremens and psychiatric co-morbidity and the interaction of delirium tremens and no psychiatric co-morbidity both decreased the probability of admission during the pandemic compared to their interaction. In other words, delirium tremens and psychiatric co-morbidity alone may have increased the probability of admission during COVID-19, but if we compare their effects to the effect of their interaction, we can see that the interaction supersedes the individual effects. Despite the limitations of our results, our present findings show the impact of the COVID-19 pandemic on the changes in AUD in a clinical setting.

The COVID-19 pandemic caused a global health crisis with several millions of infections and deaths [1]. Various effects of the pandemic have been determined and can be classified into three categories [4]. Firstly, the infection caused several millions of deaths and long-term consequences such as post-COVID syndrome. Secondly, the indirect effects of the pandemic such as the restrictions, social isolation, and disruption in health service delivery contributed to the development and worsening of psychiatric disorders [39,40]. Unemployment, financial stress, loneliness, and relationship problems all contributed to the negative effect of the pandemic and led to increased mental health risk, for example, maladaptive coping mechanisms [7,8,9,41]. Lastly, the pandemic had several socioeconomic effects that might have caused long-term psychological problems, mainly anxio-depressive symptoms [7,8,9,41].

Early studies focused on the changes in alcohol consumption during the lockdowns as a result of various mental health factors such as isolation, worry, and fear of the pandemic [23,24,25,26,27,42,43,44]. Although these studies have indicated that alcohol consumption increased during the various stages of the lockdown periods, the impact of the pandemic on the severity of AUD or on the changes in alcohol consumption in this specific population have not yet been examined in detail.

Our results revealed that the mean age, the occurrence of DT, and psychiatric co-morbidities were significantly higher among cases with a primary diagnosis of AUD during the pandemic. Interestingly, there were no significant differences in the occurrence of somatic co-morbidities and laboratory parameters between the two eras. Furthermore, our findings showed that the dates of the admissions explained the occurrence of DT.

Recent studies have revealed that somatic co-morbidities, elevated liver enzyme levels, lower levels of sodium and potassium, and decreased numbers of platelets in the serum are the most important risk factors for developing DT [31,32]. Our results indicate that there were no differences in admissions before and during the pandemic with respect to these factors. Thus, it can be suggested that the indirect effect of the pandemic—such as the restrictions, fear of infection, and the changes in health care services—may explain the increase in severe cases of AUD. The number of patient admissions and consultations for psychiatric care were lower, and the primary contact with patients was reduced for almost all patient groups with the exception of acute alcohol-related events [45]. The impact of the pandemic can be explained by various indirect factors. Most studies found that the number of depressive and anxious symptoms reported by individuals increased during and after the pandemic [46,47,48]. The exacerbation of these symptoms can raise the relapse rate among patients with AUD, potentially leading to more severe consequences.

The pandemic caused an unprecedented global crisis in the health care system and highlighted several shortcomings in the medical infrastructure [39,40]. Health care had to adapt to the new needs and expectations which caused disturbances in regular care and decreased the accessibility of non-COVID-19 patient care [39,49,50,51]. Therefore, it could be hypothesised that the lower accessibility of mental health care led to the overrepresentation of severe forms of each disorder. Furthermore, previously, it has also been demonstrated that the increase in psychological issues leads to a higher relapse rate in patients diagnosed with psychiatric disorders [5,6,7,8,9,41]. Hence, the pandemic may have an impact on the worsening or onset of DDs. However, it is important to note that the pandemic changed the criteria for hospital admission, but the changes in susceptibility of psychiatric patients and alcohol consumption may have also contributed to our results.

Our results revealed that approximately 10–30% of cases with a primary diagnosis of AUD also had secondary diagnoses of “Including symptomatic, mental disorders (F00–F09)”, “Mood (affective) disorders (F30–F39)”, organic, and “Neurotic, stress-related and somatoform disorders (F40–F48)”. Moreover, our data showed that the occurrence of these secondary conditions was higher during the pandemic. Additionally, there was a significantly higher ratio of mood and organic mental disorders during this period. Several studies have indicated that the COVID-19 pandemic had a severe impact on the development of mood disorders [9,52,53,54]. Our results support these observations and suggest the importance of co-morbid SUDs as well. Due to the indirect consequences of infection-control measures and societal changes, the pandemic increased the neuropsychiatric symptoms of patients diagnosed with organic mental disorders including dementia [55,56]. Our research extends these findings by showing a higher occurrence of these disorders in patients diagnosed with AUD in a clinical setting. However, it is important to note that the increase in psychiatric co-morbidities cannot be explained directly by the COVID-19 pandemic due to the retrospective nature of the present study.

## 5. Limitations

The present study has some limitations. First, the conclusions drawn from this study can only be generalised to a limited extent due to the retrospective collection of data from medical charts at a single regional hospital in Hungary. Second, in this study, we only analysed medical charts, so our findings only represent clinical data from a specific point in time for patients diagnosed with AUD. Third, the nature of the co-occurring mental illnesses was not revealed in detail, making it difficult to establish a direct causal relationship between the pandemic and the increased alcohol consumption among these patients. However, our findings indicate that a global health crisis like the COVID-19 pandemic can alter the clinical presentation of individuals with SUDs. Fourth, health care systems focused on admitting severe cases during the pandemic, making it difficult to determine if there was an increased incidence of DT or if these outcomes are a result of indirect effects. Nevertheless, it could be hypothesised that our findings may be explained by the changes in hospital admission criteria during the pandemic, as well as the vulnerability of patients with mental illnesses and alcohol use disorder.

## 6. Conclusions

The COVID-19 pandemic has resulted in significant health, economic, and social difficulties. Although the present study has limitations, our findings show that the pandemic had a significant impact on individuals with AUD. Additionally, our clinical observations may have implications for the future management of patients with alcohol-related issues in the post-COVID-19 era. Our findings also indicate that the pandemic may have led to an increase in the occurrence of DDs. Therefore, our clinical observations highlight the impact of the pandemic on mental health and emphasise the need for special attention to SUDs and DDs in the post-pandemic era.

## Figures and Tables

**Table 1:** Characteristics of the ‘before COVID-19’ and ‘during COVID-19’ groups.

	‘Before COVID-19’ n = 753	‘During COVID-19’ n = 329
Mean age (SD)	50.920 (11.853)	52.500 (11.567) *
Male (adj. res.)	81.7% (1.1)	78.7% (−1.1)
Female (adj. res.)	18.3% (−1.1)	21.3% (1.1)
Somatic co-morbidity (adj. res.)	65.1% (0.2)	63.8% (−0.2)
Psychiatric co-morbidity (adj. res.)	48.1% (−4.7)	63.8% (4.7) *
AWS (adj. res.)	72.7% (1.4)	68.7% (−1.4)
DT (adj. res.)	7.2% (−3.1)	13.1% (3.1) *
ARS (adj. res.)	9.8% (−1.0)	12.2% (1.0)
GGT (mean, SD)	271.757 (747.787)	361.665 (665.594)
SGOT (mean, SD)	73.150 (125.092)	75.780 (71.314)
SGPT (mean, SD)	54.64 (64.728)	54.43 (37.183)
SGOT/SGPT (mean, SD)	1.424 (0.776)	1.454 (0.818)
Sodium (mean, SD)	138.974 (4.073)	138.668 (4.508)
Potassium (mean, SD)	4.010 (0.543)	4.03 (0.639)
Platelet number (mean, SD)	215.650 (106.223)	225.570 (139.651)

Abbreviations: * = *p* < 0.05, SD = standard deviation, adj. res. = adjusted residual, AWS = alcohol withdrawal syndrome, DT = delirium tremens, ARS = alcohol-related seizure, GGT = gamma-glutamyl transferase, SGOT = serum glutamic-oxaloacetic transaminase, SGPT = serum glutamic pyruvic transaminase.

**Table 2:** Multinomial regression model of admissions during COVID-19 pandemic.

	B	SE	df	*p*	OR	95% Confidence Interval for OR	
						Lower Bound	Upper Bound
Age	0.128	0.073	1	0.080	1.137	0.985	1.311
DT^−^	−0.711	0.228	1	0.002 *	0.491	0.314	0.767
DT^+^	0		0				
Psychiatric co-morbidity−	−0.636	0.152	1	<0.001 *	0.529	0.393	0.713
Psychiatric co-morbidity+	0		0				

Abbreviations: * = *p* < 0.05, DT = delirium tremens, B = regression coefficient, SE = standard error, df = degrees of freedom, *p* = significance, OR = odds ratio.

**Table 3:** Multinomial logistic regression model of interactions between variables.

	B	SE	df	*p*	OR	95% Confidence Interval for OR	
						Lower Bound	Upper Bound
DT^−^ * Age	0.0800	0.101	1	0.430	1.083	0.888	1.321
DT^+^ * Age	0.398	0.223	1	0.074	1.488	0.962	2.302
DT^−^ * Psychiatric co-morbidity−	−1.400	0.380	1	<0.001 *	0.247	0.117	0.520
DT^−^ * Psychiatric co-morbidity+	−0.802	0.376	1	0.033 *	0.448	0.214	0.937
DT^+^ * Psychiatric co-morbidity−	−0.918	0.451	1	0.042 *	0.399	0.165	0.966
DT^+^ * Psychiatric co-morbidity+	0		0				
Psychiatric co-morbidity^−^ * Age	0.007	0.149	1	0.961	1.007	0.753	1.349
Psychiatric co-morbidity^+^ * Age	0		0				

Abbreviations: * = *p* < 0.05, DT = delirium tremens, B = regression coefficient, SE = standard error, df = degrees of freedom, *p* = significance, OR = odds ratio.

## Data Availability

The dataset of the study is available from the corresponding author (Bence András Lázár) upon request. This anonymised dataset has been generated from the registered official health insurance patient flow of the university clinic, and, due to the official data protection policy, these data are not freely available and are only available on request. Further enquiries can be directed to the corresponding author (Bence András Lázár).
